# Risk factors for breast cancer in nulliparous women

**DOI:** 10.1038/sj.bjc.6690306

**Published:** 1999-04

**Authors:** F Fioretti, A Tavani, C Bosetti, C La Vecchia, E Negri, F Barbone, R Talamini, S Franceschi

**Affiliations:** Istituto di Ricerche Farmacologiche ‘Mario Negri’, Via Eritrea 62, Milan, 20157, Italy; Istituto Statistica Medica e Biometria, Università degli Studi di Milano, Via Venezian 1, Milan, 20133, Italy; Cattedra di Igiene ed Epidemiologia, Policlinico Universitario, Università degli Studi di Udine, Via Colugna 40, Udine, 33100, Italy; Centro di Riferimento Oncologico, Via Pedemontana Occidentale, Aviano, (PN), 33081, Italy

**Keywords:** breast neoplasms, case–control studies, diet, oral contraceptives, reproductive factors, risk factors

## Abstract

The relation between hormonal and lifestyle factors and breast cancer risk in nulliparae was investigated using data from two case-control studies conducted in Italy between 1983 and 1994. The study included 1041 nulliparae with histologically confirmed incident breast cancer and 1002 nulliparous controls admitted to hospital for a wide range of acute, non-neoplastic, nonhormone-related diseases. In premenopausal nulliparae, there was an inverse relation with age at menarche [odds ratios (OR) 0.45; 95% confidence intervals (CI) 0.24–0.86 for ≥ 15 years vs < 12], while no association emerged in postmenopausal. Breast cancer risk increased with age at menopause, the OR being 1.91 (95% CI 1.26–2.90) for nulliparae reporting age at menopause ≥ 53 years compared with < 45. Abortion was not related to breast cancer risk, the OR being 0.92 for any spontaneous, 0.97 for any induced and 0.77 for ≥ 2 total abortions compared to none. The OR was 1.75 (95% CI 1.03–2.97) for women reporting their first abortion at age ≥ 30 years compared with < 30. Oral contraceptives and hormone replacement therapy in menopause were moderately related to risk. The OR was 2.71 (95% CI 1.85–3.95) in nulliparae with a family history of breast cancer and 1.60 (95% CI 1.20–2.14) in those with a history of benign breast disease. Compared with nulliparae reporting a low physical activity, the OR was 0.79 (95% CI 0.54–1.16) for those reporting intermediate/high activity. Breast cancer risk increased with total energy intake, the OR being 1.65 (95% CI 0.99–2.75) in the highest tertile; beta-carotene was inversely related to risk (OR 0.60, 95% CI 0.38–0.95) for the highest tertile. Thus, most risk factors for breast cancer in nulliparae were similar to those in women generally. © 1999 Cancer Research Campaign


*British Journal of Cancer* (1999) **79**(11/12), 1923–1928
© 1999 Cancer Research Campaign
Article no. bjoc.1998.0306

### Risk factors for breast cancer in nulliparous women

**F Fioretti[1], A Tavani[1], C Bosetti[1], C La Vecchia[1,2], E Negri[1], F Barbone[3], R Talamini[4] and S Franceschi[4]**

[1]Istituto di Ricerche Farmacologiche 'Mario Negri', Via Eritrea 62, 20157 Milan, Italy; [2]Istituto Statistica Medica e Biometria, Università degli Studi di Milano, Via Venezian 1, 20133 Milan, Italy; [3]Cattedra di Igiene ed Epidemiologia, Policlinico Universitario, Università degli Studi di Udine, Via Colugna 40, 33100 Udine, Italy; and [4]Centro di Riferimento Oncologico, Via Pedemontana Occidentale, 33081 Aviano (PN), Italy

**Summary** The relation between hormonal and lifestyle factors and breast cancer risk in nulliparae was investigated using data from two case-control studies conducted in Italy between 1983 and 1994. The study included 1041 nulliparae with histologically confirmed incident breast cancer and 1002 nulliparous controls admitted to hospital for a wide range of acute, non-neoplastic, nonhormone-related diseases. In premenopausal nulliparae, there was an inverse relation with age at menarche [odds ratios (OR) 0.45; 95% confidence intervals (CI) 0.24–0.86 for ≥ 15 years vs < 12], while no association emerged in postmenopausal. Breast cancer risk increased with age at menopause, the OR being 1.91 (95% CI 1.26–2.90) for nulliparae reporting age at menopause ≥ 53 years compared with < 45. Abortion was not related to breast cancer risk, the OR being 0.92 for any spontaneous, 0.97 for any induced and 0.77 for ≥ 2 total abortions compared to none. The OR was 1.75 (95% CI 1.03–2.97) for women reporting their first abortion at age ≥ 30 years compared with < 30. Oral contraceptives and hormone replacement therapy in menopause were moderately related to risk. The OR was 2.71 (95% CI 1.85–3.95) in nulliparae with a family history of breast cancer and 1.60 (95% CI 1.20–2.14) in those with a history of benign breast disease. Compared with nulliparae reporting a low physical activity, the OR was 0.79 (95% CI 0.54–1.16) for those reporting intermediate/high activity. Breast cancer risk increased with total energy intake, the OR being 1.65 (95% CI 0.99–2.75) in the highest tertile; beta-carotene was inversely related to risk (OR 0.60, 95% CI 0.38–0.95) for the highest tertile. Thus, most risk factors for breast cancer in nulliparae were similar to those in women generally.

**Keywords:** breast neoplasms; case–control studies; diet; oral contraceptives; reproductive factors; risk factors

There are at least three reasons for considering risk factors for breast cancer in nulliparous women. First, nulliparae are at elevated risk for breast cancer (Kelsey, 1979; Pike et al, 1983; Boyle and Leake, 1988; Kelsey and Horn-Ross, 1993; Lipworth, 1995), and any risk factor acting on nulliparae or interacting with nulliparity is relevant for individual risk assessment (Kelsey et al, 1993; Katsouyanni et al, 1997; Rothman and Greenland, 1998).

Second, restriction of analysis to nulliparae avoids the possible modifying effect or confounding from full-term pregnancy, and allows a more precise assessment of the role of other hormonal risk factors for breast cancer, such as age at menarche, menstrual cycle pattern, abortions, age at first pregnancy, use of oral contraceptives (OC) and hormone replacement therapy (HRT) in menopause (Kelsey et al, 1993; Lipworth, 1995). In particular, the relation of abortion to breast cancer risk in the absence of any modifying effect of full-term pregnancy is an open issue, as it has been suggested that abortion before full-term pregnancy is associated with increased breast cancer risk (Russo and Russo, 1980; Pike et al, 1981; Hadjimichael et al, 1986; Rosenberg et al, 1988; Howe et al, 1989).

Third, lifestyle factors which have been related to breast cancer, such as socio-cultural level, body mass index (BMI), alcohol drinking, physical activity and selected dietary habits (Willett, 1989; Hunter and Willett, 1993; Longnecker, 1994; Gammon et al, 1998; Colditz, 1998) may also be influenced by parity (Green et al, 1988), and a more precise quantification of these risk factors in nulliparae is therefore of interest.

As the information on the aetiology of breast cancer in nulliparae is sparse, we have analysed hormonal, reproductive and general lifestyle factors for breast cancer in nulliparous women using the combined data from two case–control studies conducted in Italy.

*Received 11 August 1998*
*Revised 16 October 1998*
*Accepted 19 October 1998*

*Correspondence to:* F Fioretti

### MATERIALS AND METHODS

The data were derived from two case–control studies of breast cancer, the first one conducted between January 1983 and May 1991 in the greater Milan area (La Vecchia et al, 1987), and the second one between June 1991 and February 1994 in six Italian areas: greater Milan, the province of Pordenone, the urban area of Genoa and the province of Forlì, in northern Italy; the province of Latina, in central Italy; and the urban area of Naples, in southern Italy (La Vecchia et al, 1995). The interviewers were centrally trained, and the structured questionnaires tested for reliability and reproducibility (Decarli et al, 1996; D'Avanzo et al, 1997). In both studies all interviews for cases and controls were conducted in hospital; on average, less than 4% of cases and controls approached for interview refused to participate.

The overall dataset included 5984 cases aged 22 to 80 (median age 54) and 5504 controls aged 15 to 80 (median age 55) and from these we identified 1041 nulliparous women aged 22 to 79 years (median age 56) with incident (i.e. diagnosed within the year before interview), histologically confirmed breast cancer, admitted to the major teaching and general hospitals in the areas under surveillance.

Controls were 1002 nulliparous women aged 15 to 79 years (median age 54), residing in the same geographical areas and

**Table 1** Distribution of 1041 cases of breast cancer and 1002 controls in nulliparous women, and corresponding odds ratios (OR) with 95% confidence intervals (CI), according to age, education and marital status (Italy, 1983–94)

| | Premenopausal | | | Postmenopausal | | | All |
|---|---|---|---|---|---|---|---|
| | Case | Controls | OR (95% CI)[a] | Cases | Controls | OR (95% CI)[a] | OR (95% CI)[a] |
| Age (years) | | | | | | | |
| < 40 | 128 | 263 | | 2 | 2 | | |
| 40–49 | 194 | 122 | | 25 | 26 | | |
| 50–59 | 54 | 35 | | 225 | 189 | | |
| 60–69 | – | – | | 323 | 282 | | |
| ≥ 70 | – | – | | 90 | 83 | | |
| Education (years) | | | | | | | |
| < 7 | 62 | 72 | 1[b] | 308 | 299 | 1[b] | 1[b] |
| 7–11 | 126 | 142 | 1.63 (1.03–2.56) | 203 | 164 | 1.30 (1.00–1.70) | 1.33 (1.06–1.66) |
| ≥ 12 | 188 | 206 | 2.01 (1.29–3.14) | 154 | 119 | 1.33 (0.99–1.80) | 1.56 (1.32–1.85) |
| Marital status | | | | | | | |
| Never married | 208 | 286 | 1[b] | 311 | 296 | 1[b] | 1[b] |
| Ever married | 168 | 134 | 1.29 (0.94–1.79) | 354 | 286 | 1.19 (0.95–1.50) | 1.24 (1.03–1.48) |

[a]Estimates from multiple logistic regression equations including terms for study, centre, year of recruitment, age, education and marital status. [b]Reference category.

admitted to the same network of hospitals where cases had been identified, for a wide spectrum of acute, non-neoplastic conditions unrelated to known or likely risk factors for breast cancer. Of these, 37% had traumatic conditions (mostly fractures and sprains), 28% nontraumatic orthopaedic disorders (mostly low back pain and disc disorders), 13% were admitted for acute surgical conditions (mostly abdominal, such as acute appendicitis or strangulated hernia), and 22% for miscellaneous other illnesses, such as eye, ear, nose, throat and dental disorders. Women were not included if they had been admitted for gynaecological, hormonal or neoplastic diseases.

The questionnaires included information on personal characteristics and lifestyle habits, including education, marital status and other socio-economic indicators, smoking, alcohol and coffee drinking, anthropometric variables, diet, menstrual and reproductive factors (such as age at menarche and menopause, menstrual cycles and number of abortions and births), selected medical conditions, history of lifelong use of OC and HRT, history of benign breast disease (BBD) and family history of breast cancer. Postmenopausal women were those whose menstrual period stopped within at least 1 year; the self-reported age at menopause was missing for four cases. Body mass index (BMI) was computed according to Quetelet's index (kg $m^{-2}$). The more recent study collected also information on physical activity and more detailed diet information, through a food frequency questionnaire including information on average weekly frequency of consumption of 78 foods, food groups or recipes (Decarli et al, 1996). To compute energy and selected nutrient intake, Italian food composition databases were used (Salvini et al, 1998).

### Data analysis

Odds ratios (OR) of breast cancer, and the corresponding 95% confidence intervals (CI) were derived using unconditional multiple logistic regression, fitted by the method of maximum likelihood (Breslow and Day, 1980). All the regression equations included terms for study, centre, calendar year of recruitment, quinquennia of age, education and marital status, plus further terms for menstrual, reproductive and hormonal factors, when appropriate.

### RESULTS

Table 1 gives the distribution of 1041 breast cancer cases and 1002 controls in nulliparae, and the corresponding ORs according to age, education and marital status, in separate strata of menopausal status. Compared with controls, cases were more educated, the overall OR being 1.56 for ≥ 12 years of education compared with < 7, and more often married, the overall OR being 1.24 for ever married compared with never married. The association with education was apparently stronger in premenopausal women.

Menstrual and reproductive factors are considered in Table 2. In premenopausal nulliparous women, there was a significant inverse relationship with age at menarche, with an OR of 0.45 for those reporting menarche at age ≥ 15 years compared with < 12. No significant relation emerged in postmenopausal nulliparae. No clear relation of breast cancer risk with duration of menstrual cycles was observed; however, women reporting totally irregular cycles were at nonsignificantly reduced breast cancer risk (overall OR 0.73). A significant trend in risk was observed with later age at menopause, with an OR of 1.91 among nulliparae aged ≥ 53 years at menopause compared with those aged < 45 years. Abortions were not related with breast cancer risk either in pre- or postmenopausal nulliparae. Compared with women reporting no abortion, the OR was 0.92 for any spontaneous abortion, 0.97 for any induced abortion, 1.17 for one abortion (either spontaneous or induced) and 0.77 (95% CI 0.52–1.14) for ≥ 2 abortions. Older age at first abortion was associated to breast cancer risk: the overall OR was 1.75 (95% CI 1.03–2.97) for ≥ 30 years vs < 30 at first abortion.

OC were used by 110 (12%) cases and 91 (10%) controls, and HRT by 62 (10%) cases and 46 (9%) controls. The OR was 1.62 (95% CI 1.14–2.30) for 'ever used' OC and 1.69 (95% CI 1.07–2.67) for use lasting 2 years or longer (Table 3). The OR was 1.22 (95% CI 0.81–1.84) for 'ever used' HRT, in the absence, however, of a duration–risk relationship, the OR for use lasting 2 years or longer being 1.28 (95% CI 0.66–2.45).

 *© Cancer Research Campaign 1999*

**Table 2** Distribution of 1041 cases of breast cancer and 1002 controls in nulliparous women, and corresponding odds ratios (OR) with 95% confidence intervals (CI), according to selected menstrual and obstetric factors (Italy, 1983–94)

| | Premenopausal | | | Postmenopausal | | | All |
|---|---|---|---|---|---|---|---|
| | Cases | Controls | OR (95% CI) | Cases | Controls | OR (95% CI)[a] | OR (95% CI) |
| Age at menarche (years) | | | | | | | |
| < 12 | 93 | 98 | 1[b] | 124 | 101 | 1[b] | 1[b] |
| 12 | 93 | 95 | 1.01 (0.65–1.57) | 145 | 137 | 0.84 (0.59–1.21) | 0.92 (0.70–1.21) |
| 13 | 107 | 102 | 0.94 (0.61–1.45) | 140 | 121 | 0.96 (0.66–1.38) | 0.98 (0.75–1.30) |
| 14 | 63 | 77 | 0.81 (0.51–1.31) | 127 | 119 | 0.87 (0.60–1.27) | 0.88 (0.66–1.17) |
| ≥ 15 | 20 | 48 | 0.45 (0.24–0.86) | 126 | 104 | 0.98 (0.67–1.43) | 0.84 (0.61–1.15) |
| 1 year increment | | | 0.90 (0.81–0.99) | | | 0.98 (0.92–1.04) | 0.96 (0.91–1.02) |
| $\chi^2$ trend | | | 4.46 ($P$ = 0.035) | | | | |
| Duration of menstrual periods (days) | | | | | | | |
| ≤ 25 | 60 | 56 | 0.86 (0.55–1.34) | 67 | 66 | 0.84 (0.58–1.22) | 0.88 (0.66–1.16) |
| 26–30 | 234 | 244 | 1[b] | 478 | 393 | 1[b] | 1[b] |
| ≥ 31 | 18 | 27 | 0.95 (0.48–1.87) | 34 | 19 | 1.42 (0.78–2.57) | 1.16 (0.75–1.80) |
| Irregular | 20 | 40 | 0.64 (0.34–1.18) | 27 | 24 | 0.78 (0.44–1.40) | 0.73 (0.48–1.11) |
| Unknown | 44 | 53 | | 59 | 80 | | |
| Age at menopause (years)[c] | | | | | | | |
| < 45 | | | | 100 | 130 | 1[b] | |
| 45–49 | | | | 178 | 158 | 1.49 (1.03–2.14) | |
| 50–52 | | | | 239 | 191 | 1.72 (1.19–2.48) | |
| ≥ 53 | | | | 144 | 103 | 1.91 (1.26–2.90) | |
| $\chi^2$ trend | | | | | | 9.22 ($P$ = 0.0016) | |
| Type of menopause[d] | | | | | | | |
| Natural | | | | 554 | 473 | 1[b] | |
| Artificial | | | | 111 | 109 | 1.08 (0.77–1.51) | |
| Spontaneous abortions | | | | | | | |
| No | 346 | 392 | 1[b] | 597 | 524 | 1[b] | 1[b] |
| Yes | 30 | 28 | 0.88 (0.48–1.61) | 68 | 58 | 0.98 (0.66–1.46) | 0.92 (0.66–1.28) |
| Induced abortions | | | | | | | |
| No | 345 | 393 | 1[b] | 639 | 561 | 1[b] | 1[b] |
| Yes | 31 | 27 | 1.00 (0.55–1.80) | 26 | 21 | 1.01 (0.55–1.84) | 0.97 (0.64–1.47) |
| Total number of abortions | | | | | | | |
| 0 | 316 | 369 | 1[b] | 572 | 504 | 1[b] | 1[b] |
| 1 | 38 | 24 | 1.69 (0.92–3.09) | 56 | 45 | 1.04 (0.67–1.61) | 1.17 (0.82–1.65) |
| ≥ 2 | 22 | 27 | 0.60 (0.32–1.12) | 37 | 33 | 0.94 (0.57–1.56) | 0.77 (0.52–1.14) |
| Age at first abortion (years) | | | | | | | |
| No pregnancy | 316 | 368 | 0.91 (0.47–1.76) | 572 | 504 | 1.37 (0.83–2.26) | 1.26 (0.85–1.86) |
| < 30 | 25 | 24 | 1[b] | 34 | 40 | 1[b] | 1[b] |
| ≥ 30 | 25 | 16 | 1.17 (0.46–2.94) | 51 | 28 | 2.11 (1.08–4.10) | 1.75 (1.03–2.97) |

For some variables the sum of strata does not add up to the total because of missing values. [a]Estimates from multiple logistic regression equations including terms for study, centre, year at recruitment, age, education and marital status. [b]Reference category. [c]Allowance included also terms for type of menopause. [d]Allowance included also terms for age at menopause.

Table 4 considers family history of breast cancer risk and personal history of BBD. The OR was 2.71 (95% CI 1.85–3.95) for family history in a first-degree relative and 1.60 (95% CI 1.20–2.14) for a personal history of BBD; these associations were similar in pre- and postmenopausal women.

Table 5 considers BMI, physical activity and selected dietary indicators. In premenopausal nulliparous women, a nonsignificant inverse relationship was observed with BMI: the OR was 0.82 for women whose BMI was ≥ 25 compared with < 20. In contrast, a nonsignificant direct trend in risk was observed in postmenopausal women, with an OR of 1.28 for heavier ones. Compared with women reporting a low level of physical activity, the overall OR was 0.79 (95% CI 0.54–1.16) for nulliparae reporting intermediate or high activity, with no material differences between pre- and postmenopausal women. A direct association of breast cancer risk

**Table 3** Distribution of breast cancer cases and controls in nulliparous women, and corresponding odds ratios (OR) with 95% confidence intervals (CI), according to use of oral contraceptives in all women and hormone replacement therapy in postmenopausal women (Italy, 1983–94)

| | Breast cancer | Controls | OR (95% CI)[a] |
|---|---|---|---|
| Oral contraceptive use | | | |
| Never | 931 | 911 | 1[b] |
| Ever | 110 | 91 | 1.62 (1.14–2.30) |
| Hormone replacement therapy use | | | |
| Never | 603 | 536 | 1[b] |
| Ever | 62 | 46 | 1.22 (0.81–1.84) |

[a]Estimates from multiple logistic regression equations including terms for study, centre, year of recruitment, age, education and marital status. [b]Reference category.

© Cancer Research Campaign 1999

**Table 4** Distribution of 1041 cases of breast cancer and 1002 controls in nulliparous women, and corresponding odds ratios (OR) with 95% confidence intervals (CI), according to family history of breast cancer in first degree relatives and history of benign breast disease (Italy, 1983–94)

| | Premenopausal | | | Postmenopausal | | | All |
|---|---|---|---|---|---|---|---|
| | Cases | Controls | OR (95% CI)[a] | Cases | Controls | OR (95% CI)[a] | OR (95% CI)[a] |
| Family history of breast cancer | | | | | | | |
| No | 342 | 409 | 1[b] | 584 | 552 | 1[b] | 1[b] |
| Yes | 34 | 11 | 2.86 (1.38–5.93) | 81 | 30 | 2.63 (1.69–4.09) | 2.71 (1.85–3.95) |
| History of benign breast disease | | | | | | | |
| No | 305 | 382 | 1[b] | 570 | 530 | 1[b] | 1[b] |
| Yes | 71 | 38 | 1.69 (1.06–2.68) | 95 | 52 | 1.63 (1.13–2.37) | 1.60 (1.20–2.14) |

[a]Estimates from multiple logistic regression equations including terms for study, centre, year at recruitment, age, education, marital status, age at menarche, age at menopause, oral contraceptive and hormone replacement therapy use. [b]Reference category.

**Table 5** Distribution of 1041 cases of breast cancer and 1002 controls in nulliparous women, and corresponding odds ratios (OR) with 95% confidence intervals (CI)[a], according to body mass index, physical activity and selected dietary factors (Italy, 1983–94)

| | Premenopausal | | | Postmenopausal | | | All |
|---|---|---|---|---|---|---|---|
| | Cases | Controls | OR (95% CI) | Cases | Controls | OR (95% CI) | OR (95% CI) |
| Body mass index (kg $m^{-2}$) | | | | | | | |
| < 20 | 97 | 124 | 1[b] | 83 | 85 | 1[b] | 1[b] |
| 20–24 | 204 | 224 | 0.88 (0.60–1.28) | 322 | 281 | 1.19 (0.84–1.71) | 1.02 (0.79–1.32) |
| ≥ 25 | 75 | 72 | 0.82 (0.50–1.35) | 258 | 216 | 1.28 (0.88–1.86) | 1.08 (0.81–1.43) |
| Physical activity[c] | | | | | | | |
| Low | 50 | 51 | 1[b] | 54 | 39 | 1[b] | 1[b] |
| Intermediate/high | 91 | 97 | 0.85 (0.46–1.56) | 206 | 193 | 0.74 (0.44–1.24) | 0.79 (0.54–1.16) |
| Total energy intake (kcal $day^{-1}$)[c] | | | | | | | |
| < 1511.3 | 26 | 32 | 1[b] | 76 | 95 | 1[b] | 1[b] |
| 1511.3–1953 | 54 | 49 | 1.52 (0.66–3.53) | 106 | 78 | 1.91 (1.13–3.23) | 1.65 (1.08–2.54) |
| > 1953 | 61 | 67 | 1.36 (0.52–3.52) | 78 | 59 | 1.72 (0.91–3.23) | 1.65 (0.99–2.75) |
| $\chi^2$ trend | | | 0.19 ($P$ = 0.66) | | | 2.78 ($P$ = 0.095) | 3.25 ($P$ = 0.07) |
| Alcohol drinking (drinks $day^{-1}$) | | | | | | | |
| Nondrinkers | 126 | 172 | 1[b] | 183 | 182 | 1[b] | 1[b] |
| 0.1–1 | 129 | 133 | 1.04 (0.72–1.51) | 203 | 189 | 1.07 (0.80–1.44) | 1.05 (0.83–1.31) |
| > 1 | 120 | 114 | 0.90 (0.61–1.33) | 279 | 209 | 1.23 (0.93–1.63) | 1.12 (0.89–1.40) |
| Beta-carotene intake (µg $day^{-1}$)[c] | | | | | | | |
| < 3165.5 | 40 | 46 | 1[b] | 93 | 81 | 1[b] | 1[b] |
| 3165.5–5028.3 | 62 | 44 | 1.54 (0.74–3.19) | 90 | 82 | 0.75 (0.46–1.23) | 0.97 (0.65–1.43) |
| > 5028.3 | 39 | 58 | 0.68 (0.29–1.59) | 77 | 69 | 0.53 (0.29–0.96) | 0.60 (0.38–0.95) |
| $\chi^2$ trend | | | 1.04 ($P$ = 0.47) | | | 4.45 ($P$ = 0.035) | 4.66 ($P$ = 0.031) |

For some variables the sum of strata does not add up to the total because of missing values. [a]Estimates from multiple logistic regression equations including terms for study, centre, year at recruitment, age, education, marital status, age at menopause, oral contraceptive and hormone replacement therapy use and all the above variables. [b]Reference category. [c]Based on 401 cases and 308 controls (second study only).

with total energy intake was observed: the OR for the highest tertile of intake compared with the lowest one was 1.36 (95% CI 0.52–3.52) among premenopausal women and 1.72 (95% CI 0.91–3.23) among postmenopausal women (overall OR 1.65, 95% CI 0.99–2.75). No apparent relation was observed between total alcohol intake and breast cancer risk in premenopausal nulliparae, while a nonsignificant elevated risk was found in postmenopausal (OR of 1.23 for drinkers of > 1 drink $day^{-1}$ compared to nondrinkers). The intake of beta-carotene – a nonspecific indicator of fruit and vegetables – was inversely related to breast cancer risk, with an OR of 0.68 (95% CI 0.29–1.59) in premenopausal women and 0.53 (95% CI 0.29–0.96) in postmenopausal women for the highest tertile of intake.

### DISCUSSION

The present study indicates that most recognized risk factors for breast cancer also operate in nulliparous women. These include education, age at menopause, OC, HRT, family history of breast cancer, personal history of BBD, total energy intake and BMI in postmenopausal women; moderate inverse relation emerged with age at menarche in premenopausal women, irregular menstrual cycles, physical activity and beta-carotene intake (Trichopoulos et al, 1972; Pike et al, 1983; Willett, 1989; Block et al, 1992; Hunter and Willett 1993; Parazzini et al, 1993; Longnecker, 1994; Collaborative Group on Hormonal Factors in Breast Cancer, 1996, 1997; Gammon et al, 1998).

 *© Cancer Research Campaign 1999*

A strength of this study is that it included over 1000 nulliparous cases and 1000 controls, and was therefore large enough to provide reasonably stable risk estimates. Most estimates were consistent with available knowledge of breast cancer epidemiology, indicating that it is unlikely that parity is a major modifying factor of breast carcinogenesis.

Most other strengths and limitations of this study are common to hospital-based case–control studies (Mantel and Haenszel, 1959; Breslow and Day, 1980). Although this study was not population-based, cases were identified in the major teaching and general hospitals of the area under surveillance, the participation of cases and controls was almost complete, and the hospital-based design may improve the comparability of recall of several covariates by cases and controls (Breslow and Day, 1980; D'Avanzo et al, 1996, 1997). Only acute conditions, unrelated to known or potential risk factors for breast cancer were included in the comparison group. Some fractures in older women are hormonally related; however, separate comparison of cases with major diagnostic categories of controls (traumas, other orthopaedic, other diseases) produced comparable results. Reproducibility and validity of the questionnaire was satisfactory (Franceschi et al, 1995; D'Avanzo et al, 1996, 1997; Decarli et al, 1996). The results were similar in the two studies (La Vecchia et al, 1987; Talamini et al, 1996), confirming their consistency. Further, adjustment for education, lifestyle and menstrual characteristics did not appreciably modify any risk estimate.

A major limitation of the present study is the absence of information on the reasons for nulliparity, whether infertility or avoidance of pregnancy A proxy indicator of this difference is given by marital status, an excess of married women among cases pointing to a possible role of subfertility. However, the main findings were consistent for ever married and never married women. Moreover, no consistent association has been reported between breast cancer risk and history of medically diagnosed infertility (Weiss et al, 1998).

Of specific interest is the absence of any association of breast cancer risk with spontaneous or induced abortion. Experimental data in rodents suggested that abortion before a full-term pregnancy increased the yield of breast neoplasms (Russo and Russo, 1980) and epidemiological support to a specific role of abortions before first birth came from a case–control study of women aged 32 years or younger at diagnosis (Pike et al, 1981). Epidemiological data on the relation of abortion with breast cancer risk in the general population are controversial (Daling et al, 1994, 1996; Brind et al, 1996; Newcomb et al, 1996; Rookus and van Leeuwen, 1996), although the available evidence does not indicate a major association in the general population or in nulliparae (Lipworth et al, 1995; Michels et al, 1995; Tavani et al, 1996; Melbye et al, 1997; Palmer et al, 1997; Wingo et al, 1997). The lack of association in this study does not support the hypothesis that an abortion before (or in absence of) the maturation of the breast tissue induced by a full-term pregnancy plays a role in the process of breast carcinogenesis (Boyle and Leake, 1988; Key and Beral, 1992). Compared with women aged less than 30 years at first abortion, the OR was elevated in those reporting their first abortion at age ≥ 30, suggesting that age at either full-term or incomplete pregnancy may have a similar influence on breast cancer risk (MacMahon et al, 1970; Lipworth et al, 1995; Michels et al, 1995; Decarli et al, 1996; Melbye et al, 1997; Wingo et al, 1997).

The associations with OC and HRT are consistent with the results of systematic reanalyses of individual data (Collaborative Group on Hormonal Factors in Breast Cancer, 1996, 1997). We were unable to estimate risk for current female hormone users because of the low prevalence of use among Italian women. We found, however, a slightly stronger association for both OC and HRT. These differences may be due to chance, as in our population OC and HRT use was relatively rare, or to some unavailable correlate of oestrogen hormone use in nulliparous women in Italy.

It is, moreover, increasingly clear that certain well-recognized risk factors, including high educational attainment operate beyond their reproductive correlates and may be indicators of high risk at early age. In our study, age at menarche and educational attainment seemed to exert more influence in premenopausal nulliparous women.

In this (La Vecchia et al, 1987, Talamini et al, 1996) and in most other studies (Kelsey, 1979; Kelsey and Horn-Ross, 1993; Lipworth, 1995; Weiss et al, 1998) nulliparous women were at elevated breast cancer risk compared with parae. The similar magnitude of associations in nulliparous compared with parous women for a number of modifiable risk factors may have implications in relation to preventive measures (Katsouyanni et al, 1997; Rothman and Greenland, 1998), because intervention in these factors, such as dietary factors and physical activity may have a greater impact on breast cancer risk for nulliparous women.

### ACKNOWLEDGEMENTS

This work was conducted with the contribution of the Italian Association for Cancer Research. The authors wish to thank Mrs Judy Baggott, Ms M Paola Bonifacino and the GA Pfeiffer Memorial Library staff for editorial assistance.

### REFERENCES

Block G, Patterson B and Subar A (1992) Fruit, vegetables, and cancer prevention: a review of the epidemiological evidence. *Nutr Cancer* **18**: 1–29

Boyle P and Leake R (1988) Progress in understanding breast cancer: epidemiological and biological interactions. *Breast Cancer Res Treatment* **11**: 91–112

Breslow NE and Day NE (1980) *Statistical Methods in Cancer Research. Vol. 1. The analysis of case-control studies*. IARC Sci Publ 32

Brind J, Chinchilli VM, Severs WB and Summy-Long J (1996) Induced abortion as an independent risk factor for breast cancer: a comprehensive review and meta-analysis. *J Epidemiol Community Health* **50**: 481–496

Colditz GA (1998) Relationship between estrogen levels, use of hormone replacement therapy, and breast cancer. *J Natl Cancer Inst* **90**: 814–823

Collaborative Group on Hormonal Factors in Breast Cancer (1996) Breast cancer and hormonal contraceptives: collaborative reanalysis of individual data on 53 297 women with breast cancer and 100 239 women without breast cancer from 54 epidemiological studies. *Lancet* **347**: 1713–1727

Collaborative Group on Hormonal Factors in Breast Cancer (1997) Breast cancer and hormone replacement therapy: collaborative reanalysis of data from 51 epidemiological studies of 52 705 women with breast cancer and 108 411 women without breast cancer. *Lancet* **350**: 1047–1059

D'Avanzo B, La Vecchia C, Katsouyanni K, Negri E and Trichopoulos D (1996) Reliability of information on cigarette smoking and beverage consumption provided by hospital controls. *Epidemiology* **7**: 312–315

D'Avanzo B, La Vecchia C, Katsouyanni K, Negri E and Trichopoulos D (1997) An assessment, and reproducibility of food frequency data provided by hospital controls. *Eur J Cancer Prev* **6**: 288–293

Daling JR, Brinton LA, Voight LF, Weiss NS, Coates RJ, Malone KE, Schoenberg JB and Gammon M (1996) Risk of breast cancer among white women following induced abortion. *Am J Epidemiol* **144**: 373–380

Daling JR, Malone KE, Voigt LF, White E and Weiss NS (1994) Risk of breast cancer among young women: relationship to induced abortion. *J Natl Cancer Inst* **86**: 1584–1592

Decarli A, Franceschi S, Ferraroni M, Gnagnarella P, Parpinel MT, La Vecchia C, Negri E, Salvini S, Falcini F and Giacosa A (1996) Validation of a food

© Cancer Research Campaign 1999 

frequency questionnaire to assess dietary intakes in cancer studies in Italy: results for specific nutrients. *Ann Epidemiol* **6**: 110–118

Franceschi S, Barbone F, Negri E, Decarli A, Ferraroni M, Filiberti R, Giacosa A, Gnagnarella P, Nanni O, Salvini S and La Vecchia C (1995) Reproducibility of an Italian food frequency questionnaire for cancer studies. Results for specific nutrients. *Ann Epidemiol* **5**: 69–75

Gammon MD, John EM and Britton JA (1998) Recreational and occupational physical activities and risk of breast cancer. *J Natl Cancer Inst* **90**: 100–117

Green A, Beral V and Moser K (1988) Mortality in women in relation to their childbearing history. *Br Med J* **297**: 391–395

Hadjimichael OC, Boyle CA and Meigs JW (1986) Abortion before first livebirth and risk of breast cancer. *Br J Cancer* **53**: 281–284

Howe H, Senie RT, Bzduch H and Herzfeld P (1989) Early abortion and breast cancer risk among women under age 40. *Int J Epidemiol* **18**: 300–304

Hunter DJ and Willett WC (1993) Diet, body size, and breast cancer. *Epidemiol Rev* **15**: 110–131

Katsouyanni K, Signorello LB, Lagiou P, Egan K and Trichopoulos D (1997) Evidence that adult life risk factors influence the expression of familial propensity to breast cancer. *Epidemiology* **8**: 592–595

Kelsey JL (1979) A review of the epidemiology of breast cancer. *Epidemiol Rev* **1**: 74–109

Kelsey JL, Gammon MD and John EM (1993) Reproductive factors and breast cancer. *Epidemiol Rev* **15**: 36–47

Kelsey JL and Horn-Ross PL (1993) Breast cancer: magnitude of the problem and descriptive epidemiology. *Epidemiol Rev* **15**: 7–16

Key TJA and Beral V (1992) Sex hormones and cancer. In *Mechanisms of Carcinogenesis in Risk Identification*. Vainio H, Magee PN, McGregor DB, McMichael AJ (eds), pp 255–269. IARC: Lyon

La Vecchia C, Decarli A, Parazzini F, Gentile A, Negri E, Cecchetti G and Franceschi S (1987) General epidemiology of breast cancer in Northern Italy. *Int J Epidemiol* **16**: 347–355

La Vecchia C, Negri E, Franceschi S, Talamini R, Amadori D, Filiberti R, Conti E, Montella M, Veronesi A, Parazzini F, Ferraroni M and Decarli A (1995) Oral contraceptives and breast cancer: a cooperative Italian study. *Int J Cancer* **60**: 163–167

Lipworth L (1995) Epidemiology of breast cancer. *Eur J Cancer Prev* **4**: 7–30

Lipworth L, Katsouyanni K, Ekbom A, Michels KB and Trichopoulos D (1995) Abortion and the risk of breast cancer: a case-control study in Greece. *Int J Cancer* **61**: 181–184

Longnecker MP (1994) Alcoholic beverage consumption in relation to risk of breast cancer: meta-analysis and review. *Cancer Causes Control* **5**: 73–82

MacMahon B, Cole P, Lin TM, Lowe CR, Mirra AP, Ravnihar B, Salber EJ, Valaoras VG and Yuasa S (1970) Age at first birth and breast cancer risk. *Bull WHO* **43**: 209–221

Mantel N and Haenszel W (1959) Statistical aspects of the analysis of data from retrospective studies of disease. *J Natl Cancer Inst* **22**: 719–748

Melbye M, Wohlfahrt J, Olsen JH, Frisch M, Westergaard T, Helweg-Larsen K and Andersen PK (1997) Induced abortion and the risk of breast cancer. *N Engl J Med* **336**: 81–85

Michels KB, Hsieh C-c, Trichopoulos D and Willett WC (1995) Abortion and cancer risk in seven countries. *Cancer Causes Control* **6**: 75–82

Newcomb PA, Storer BE, Longnecker MP, Mittendorf R, Greenberg ER and Willett WC (1996) Pregnancy termination in relation to risk of breast cancer. *JAMA* **275**: 283–287

Palmer JR, Rosenberg L, Rao RS, Zauber A, Strom BL, Warshauer ME, Stolley PD and Shapiro S (1997) Induced and spontaneous abortion in relation to risk of breast cancer (United States). *Cancer Causes Control* **8**: 841–849

Parazzini F, La Vecchia C, Negri E, Franceschi S and Tozzi L (1993) Lifelong menstrual pattern and risk of breast cancer. *Oncology* **50**: 222–225

Pike MC, Henderson BE, Casagrande JT, Rosario I and Gray GE (1981) Oral contraceptive use and early abortion as risk factors for breast cancer in young women. *Br J Cancer* **43**: 72–76

Pike MC, Krailo MD and Henderson BE (1983) Hormonal risk factors, ‘breast tissue age’ and the age incidence of breast cancer. *Nature* **303**: 767–770

Rookus MA and van Leeuwen FE (1996) Induced abortion and risk for breast cancer: reporting (recall) bias in a Dutch case-control study. *J Natl Cancer Inst* **88**: 1759–1764

Rosenberg L, Palmer JR, Kaufman DW, Strom BL, Schottenfeld D and Shapiro S (1988) Breast cancer in relation to the occurrence and time of induced and spontaneous abortion. *Am J Epidemiol* **127**: 981–989

Rothman KJ and Greenland S (1998) *Modern Epidemiology*, 2nd edn. Lippincott-Raven: Philadelphia

Russo J and Russo IH (1980) Susceptibility of the mammary gland to carcinogenesis. II. Pregnancy interruption as a risk factor in tumor incidence. *Am J Pathol* **100**: 497–512

Salvini S, Parpinel M, Gnagnarella P, Maisonneuve P and Turrini A (eds) (1998) *Banca Dati di Composizione degli Alimenti per Studi Epidemiologici in Italia*. Istituto Europeo di Oncologia: Milano

Talamini R, Franceschi S, La Vecchia C, Negri E, Borsa L, Montella M, Falcini F, Conti E and Rossi C (1996) The role of reproductive and menstrual factors in cancer of the breast before and after menopause. *Eur J Cancer* **32A**: 303–310

Tavani A, La Vecchia C, Franceschi S, Negri E, D’Avanzo B and Decarli A (1996) Abortion and breast cancer risk. *Int J Cancer* **65**: 401–405

Trichopoulos D, MacMahon B and Cole P (1972) Menopause and breast cancer risk. *J Natl Cancer Inst* **48**: 605–613

Weiss HA, Troisi R, Rossing MA, Brogan D, Coates RJ, Gammon MD, Potischman N, Swanson CA and Brinton LA (1998) Fertility problems and breast cancer risk in young women: a case-control study in the United States. *Cancer Causes Control* **9**: 331–339

Willett W (1989) The search for the causes of breast and colon cancer. *Nature* **338**: 389–394

Wingo PA, Newsome K, Marks JS, Calle EE and Parker SL (1997) The risk of breast cancer following spontaneous or induced abortion. *Cancer Causes Control* **8**: 93–108

 *© Cancer Research Campaign 1999*
